# Impact of repetitive negative thinking on subjective cognitive decline: insights into cognition and brain structure

**DOI:** 10.3389/fnagi.2024.1441359

**Published:** 2024-08-13

**Authors:** Lídia Mulet-Pons, Cristina Solé-Padullés, María Cabello-Toscano, Kilian Abellaneda-Pérez, Ruben Perellón-Alfonso, Gabriele Cattaneo, Javier Solana Sánchez, Vanessa Alviarez-Schulze, Nuria Bargalló, Josep M. Tormos-Muñoz, Alvaro Pascual-Leone, David Bartrés-Faz, Lídia Vaqué-Alcázar

**Affiliations:** ^1^Department of Medicine, Faculty of Medicine and Health Sciences, Institute of Neurosciences, University of Barcelona, Barcelona, Spain; ^2^Institut d’Investigacions Biomèdiques August Pi i Sunyer (IDIBAPS), Barcelona, Spain; ^3^Guttmann Institute, Institut Universitari de Neurorehabilitació, affiliated to the Autonomous University of Barcelona, Badalona, Spain; ^4^Fundació Institut d’Investigació en Ciències de la Salut Germans Trias i Pujol, Badalona, Spain; ^5^Neuroradiology Section, Department of Radiology, Diagnostic Image Center, Hospital Clinic of Barcelona, University of Barcelona, Barcelona, Spain; ^6^Centro de Investigación Traslacional San Alberto Magno, Universidad Católica de Valencia San Vicente Mártir, Valencia, Spain; ^7^Hinda and Arthur Marcus Institute for Aging Research, Deanna and Sidney Wolk Center for Memory Health, Harvard Medical School, Hebrew SeniorLife, Boston, MA, United States; ^8^Department of Neurology, Harvard Medical School, Boston, MA, United States; ^9^Sant Pau Memory Unit, Department of Neurology, Institut d’Investigacions Biomèdiques Sant Pau-Hospital de Sant Pau, Universitat Autònoma de Barcelona, Barcelona, Spain

**Keywords:** aging, subjective cognitive decline, rumination, neuroimaging, cognition, repetitive negative thinking, risk factors

## Abstract

**Introduction:**

Individuals with subjective cognitive decline (SCD) express concern about self-perceived cognitive decline despite no objective impairment and are at higher risk of developing Alzheimer’s disease. Despite documented links between SCD and repetitive negative thinking (RNT), the specific impact of RNT on brain integrity and cognition in exacerbating the SCD condition remains unclear. We aimed to investigate the influence of RNT on global cognition and brain integrity, and their interrelationships among healthy middle-aged and older adults experiencing SCD.

**Methods:**

Out of 616 individuals with neuroimaging and neuropsychological data available, 89 (mean age = 56.18 years; 68.54% females) met SCD criteria. Eighty-nine non-SCD individuals matched by age, sex, and education were also selected and represented the control group (mean age = 56.09 years; 68.54% females). Global cognition was measured using the preclinical Alzheimer’s cognitive composite (PACC5), which includes dementia screening, episodic memory, processing speed, and category fluency tests. RNT was calculated through three questionnaires assessing intrusive thoughts, persistent worry, and rumination. We generated cortical thickness (CTh) maps and quantified the volume of white matter lesions (WML) in the whole brain, as grey and white matter integrity measures, respectively.

**Results:**

SCD individuals exhibited higher RNT scores, and thinner right temporal cortex compared to controls. No differences were observed in PACC5 and WML burden between groups. Only the SCD group demonstrated positive associations in the CTh-PACC5, CTh-RNT, and WML-RNT relationships.

**Discussion:**

In this cross-sectional study, RNT was exclusively associated with brain integrity in SCD. Even though our findings align with the broader importance of investigating treatable psychological factors in SCD, further research may reveal a modulatory effect of RNT on the relationship between cognition and brain integrity in SCD.

## 1 Introduction

The global rise in dementia prevalence poses a concerning public health challenge (Prince et al., 2016), underscoring the urgent need for comprehensive strategies to address its impact on healthcare systems worldwide. Remarkably, approximately 40% of global dementia, the leading cause of which is Alzheimer’s disease (AD), could potentially be prevented or delayed by addressing modifiable factors encompassing lifestyles across the lifespan (Livingston et al., 2020). The impact of lifestyle factors on inter-individual differences in cognitive aging and susceptibility to clinical dementia has been described by the theory of cognitive reserve and resilience-related processes (Stern et al., 2020, 2023). Complementarily, Marchant and Howard (2015) proposed the cognitive debt hypothesis, suggesting that cumulative cognitive deficits resulting from various psychological risk factors may deplete cognitive reserve (e.g., depression and anxiety symptoms, distress, neuroticism, sleep disorders, and life stressors), thereby increasing vulnerability to AD and leading to diminished cognitive functioning over time. Repetitive negative thinking (RNT) is a modifiable and central transdiagnostic construct within the cognitive debt hypothesis due to its applicability across various conditions. It is defined by self-relevant persistent negative thoughts that activate cognitive representations of previous (i.e., past-directed rumination) or anticipated (i.e., future-directed worry) stressful events (McLaughlin et al., 2007). RNT prolongs stress physiological responses (Brosschot et al., 2006) and has been independently related to amyloid and tau deposition, as well as cognitive decline in healthy adults in the study by Marchant et al. (2020). Notably, Schlosser et al. (2020) found that RNT better differentiated subjective cognitive decline (SCD) classification in older adults than other psychological factors, such as purpose in life or personality traits.

SCD refers to individuals experiencing a worsening in cognitive function compared to a previously normal status, without objective cognitive impairment in a neuropsychological assessment (Jessen et al., 2014). Although SCD has been regarded as a heterogeneous condition, research indicates that individuals with SCD have an increased risk of developing dementia compared with those without SCD (Slot et al., 2018). This has led to the acknowledgment of SCD as a preclinical stage in the AD continuum, potentially indicating the onset of neurodegenerative and cerebrovascular changes silently occurring years before the manifestation of clinical symptoms and formal dementia diagnoses (Bateman et al., 2012; Jack, 2022). Moreover, higher rates of worrying about cognitive complaints impacted the odds ratio of developing AD prodromal stages such as mild cognitive impairment (MCI) (Pike et al., 2022). However, while the association between SCD and RNT has been previously reported (Schlosser et al., 2020; Solé-Padullés et al., 2022), the specific influence of RNT levels on brain integrity and cognition in this condition remains to be elucidated.

Reliable indicators of cerebral status in SCD are cortical thickness (CTh) and the volume of white matter lesions (WML), which can be assessed through structural magnetic resonance imaging (MRI), reflecting grey and white matter integrity, respectively (see revisions in MacDonald and Pike, 2021). Notably, individuals with SCD exhibit thinner temporal and parietal cortices (Verfaillie et al., 2016), and a greater global WML burden (Benedictus et al., 2015). Research has shown that RNT exhibits both positive and negative associations with CTh in brain regions involved in cognitive control and emotional processing (see review by Demnitz-King et al., 2021), particularly in the prefrontal and cingulate regions (Bush et al., 2000; Dixon et al., 2017). However, Demnitz-King et al. (2021) argued in their review that the direction of these CTh associations and the specific affected regions by RNT depend on the methods used to assess this metric and the clinical status of the sample. Moreover, in the same review, RNT has been negatively associated with white matter microstructure alterations of several long-range fasciculi. In addition, Marchant and Howard (2015) review and Marchant et al. (2020) empirical paper highlight RNT as a risk factor for AD, showing its association with brain disintegration due to AD pathology depositions. Hence, given these associations between RNT and both grey and white matter integrity, it becomes relevant to explore the intersection between RNT and SCD, particularly how RNT may impact brain integrity in individuals with SCD, where these effects may be particularly pronounced. Furthermore, investigating the correlates of grey and white matter in SCD compared to controls may provide insights into clarifying the structural network underlying RNT.

In summary, albeit SCD may represent a risk for a preclinical phase of dementia, and although RNT may be a prominent feature among SCD, little is known regarding how this psychological process may contribute to cognitive debt in terms of brain integrity in the SCD condition compared to a control group. Within this context, in the present cross-sectional study, our aim was (1) to characterize differences in global cognition, RNT, and brain integrity (i.e., CTh and WML) between individuals with SCD and a matched control group; (2) to examine the association between global cognition and brain integrity in our groups; and (3) to study the potential influence of RNT levels on global cognition, brain integrity and the relationship between these variables by group (SCD vs. control). We hypothesize that SCD individuals will exhibit significantly higher levels of RNT, as well as lower brain integrity and global cognition compared to the control group. Also, in the context of expected negative associations between global cognition and brain integrity, SCD individuals with higher levels of RNT might have a stronger impact on the relationships previously identified.

## 2 Materials and methods

### 2.1 Participants

**Figure 1:**
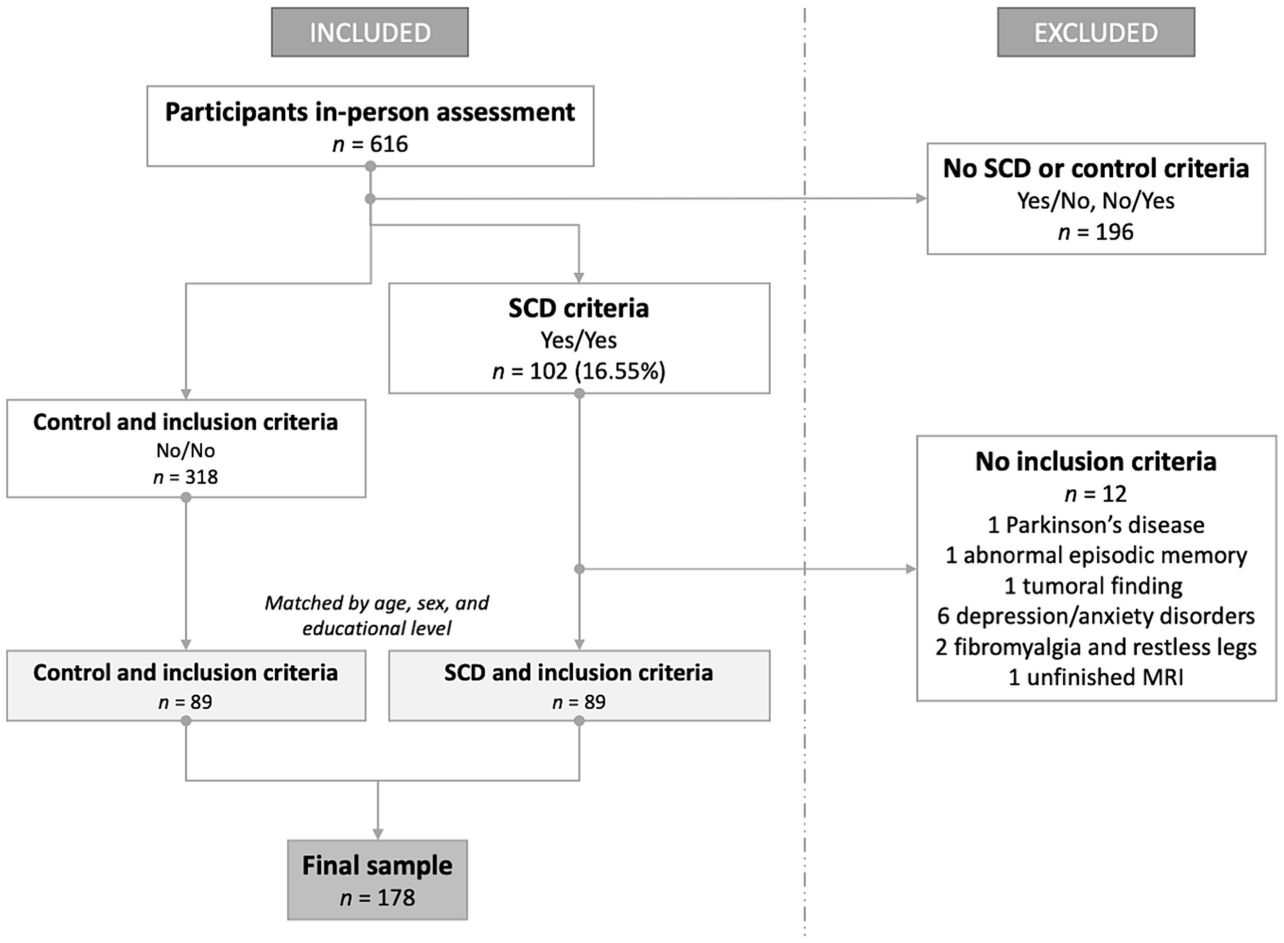
Flowchart of sample formation depending on inclusion and exclusion criteria. SCD, subjective cognitive decline; MRI, magnetic resonance imaging.

Volunteers from the community-based Barcelona Brain Health Initiative (BBHI) (Cattaneo et al., 2018) cohort were assessed for SCD criteria with statements like “In the last 2 or 3 years my memory has worsened considerably” and “I am worried about the state of my memory” with “Yes”/”No” answers. A total of 616 participants answered both statements and underwent in-person medical, cognitive, and MRI assessments (Cattaneo et al., 2020). First, we defined individuals as having SCD if they answered “Yes” to both statements (Jessen et al., 2020), and classified individuals as controls if they answered “No” to both statements. Overall, 102 individuals met the SCD criteria (prevalence of 16.55% in the entire cohort), consistent with previous studies involving comparable community-based samples (Hao et al., 2017; Taylor et al., 2018). Second, inclusion criteria were applied as explained elsewhere (Mulet-Pons et al., 2023), including 89 out of 102 in the SCD group (see Figure 1). For the control group, we selected a demographically matched group of 89 individuals among the 318 participants who answered “No” in both SCD statements and met the inclusion criteria (see Statistical analyses section for further details). As a result, 178 participants formed our analysis sample.

All study procedures were approved by the Institutional Review Board (IRB 00003099 at the University of Barcelona) and Comité d’Ètica i Investigació Clínica de la Unió Catalana d’Hospitals under the Code of Ethics of the World Medical Association (Declaration of Helsinki). Written informed consent was obtained from each participant before study enrollment.

### 2.2 Psychological and cognitive measures

As in previous studies (Schlosser et al., 2020; Solé-Padullés et al., 2022), RNT was assessed using three questionnaires of intrusive thoughts, persistent worry, and rumination. Firstly, the Perseverative Thinking Questionnaire (PTQ) was administered for the intrusive thoughts, consisting of 15 items with a 5-point Likert scale validated in Spanish (Ruiz et al., 2020) based on the original version (Ehring et al., 2011). Worry was measured by the Penn State Worry Questionnaire (PSWQ) comprising 8 items with a 5-point Likert scale (Hopko et al., 2003; Spanish version by Nuevo et al., 2007). Finally, the Rumination Responses Scale (RRS) was employed to evaluate rumination using the Spanish version by Hervás (2008), including 22 items in a 4-point Likert scale. A composite score for RNT was calculated by summing the previously standardized values (i.e., z-scored) of the three questionnaires, as done elsewhere (higher z-scores indicating higher RNT) (Solé-Padullés et al., 2022).

A global cognition composite score sensitive to detecting preclinical AD-related decline was obtained using the Preclinical Alzheimer’s Cognitive Composite 5 (PACC5) (Donohue et al., 2014). An abridged version of the PACC5 was computed based on the original one, including a screening of dementia, episodic memory, attention scores, and category fluency domain (Papp et al., 2017). The tests included in our composite were the MMSE, delayed recall of the Rey Auditory Verbal Learning Test (RAVLT), the total score of the Symbol Digit Modalities Test (SDMT), and the total score of category fluency for animals, respectively. We separately obtained the z-score for each test measure and then calculated the unweighted average.

### 2.3 MRI acquisition and preprocessing

MRI data were acquired in a 3 T Siemens scanner (MAGNETOM Prisma) with a 32-channel head coil at the Unitat d’Imatge per Ressonància Magnètica IDIBAPS (Institut d’Investigacions Biomèdiques August Pi i Sunyer) at Hospital Clínic de Barcelona, Barcelona. Automated preprocessing of structural T1-weighted images (0.8-mm isotropic voxel) and CTh maps were generated using FreeSurfer (version 6.0) (Fischl et al., 2002).^1^ Before the statistical analysis, CTh maps were smoothed using a 2D Gaussian kernel of 15 mm full width at half maximum (FWHM). The total WML burden of the whole brain was quantified as the total volume (in mm^3^) of all voxels identified as white matter hypointensities in the standard space adjusted for the estimated total intracranial volume. See Supplementary material for further details about MRI acquisition parameters, preprocessing, vertex-wise analyses, and MRI-derived values extraction.

### 2.4 Statistical analyses

Statistical analyses were conducted using RStudio [version 2023.6.2.561, RStudio Team (2020)]^2^ and FreeSurfer (version 6.0; see text footnote 1, respectively). All results were considered significant with a level of confidence of 95%, resulting in an alpha (*p*-value) of 0.05.

The control group was matched using the MatchIt package (Ho et al., 2011)^3^ of Rstudio (v. 4.2.1 Rstudio Team, 2020).^4^ We paired our SCD and control group by age, sex, and level of education to control demographic influences in our statistical analyses using the “nearest” method which selected the control participants closest in terms of our covariates to each SCD participant.

Then, we assessed the normality of our variables using the Shapiro–Wilk test. The RNT, PACC5, and WML variables were not normally distributed (*p* < 0.05) and given the size of our groups (<100) we performed the non-parametric Wilcoxon and Kruskal–Wallis tests for group comparisons (SCD vs. control) as respects our first aim of the study.

Regarding our second and third aims, we employed multivariate GLMs to display pairwise relationships for the entire sample among our variables of interest (PACC5, RNT, CTh, and WML), along with a group interaction element to evaluate whether there were any group differences in the slope of these associations. Furthermore, to underscore the significance of our findings and shed further light on our research focus, we conducted exploratory analyses to investigate the influence of RNT levels (lowRNT-SCD and highRNT-SCD) within the SCD condition on the CTh and WML correlates (see Supplementary material for further details).

The GLMs were adjusted by age, sex, and educational level to control the impact of demographics in the pairwise relationships tested, while we did not control for demographics in group differences analyses because the matched method used already accounted for this. Model assumptions were checked using Q-Q plots and fitted versus residual plots, and the normality of the residuals was checked using Shapiro–Wilk. Also, we controlled outlier influences by Cook’s distance analyses.

## 3 Results

### 3.1 Demographics and group differences

Our sample consisted of 178 participants with a mean age of 56.13 years (± 7.72, range: 42–72 years). Females constituted 68.54% of the sample, while 69.10% of participants reported a tertiary educational level. Descriptive statistics for the entire sample, as well as for the SCD and control groups, are provided in Supplementary Table S1.

**Figure 2:**
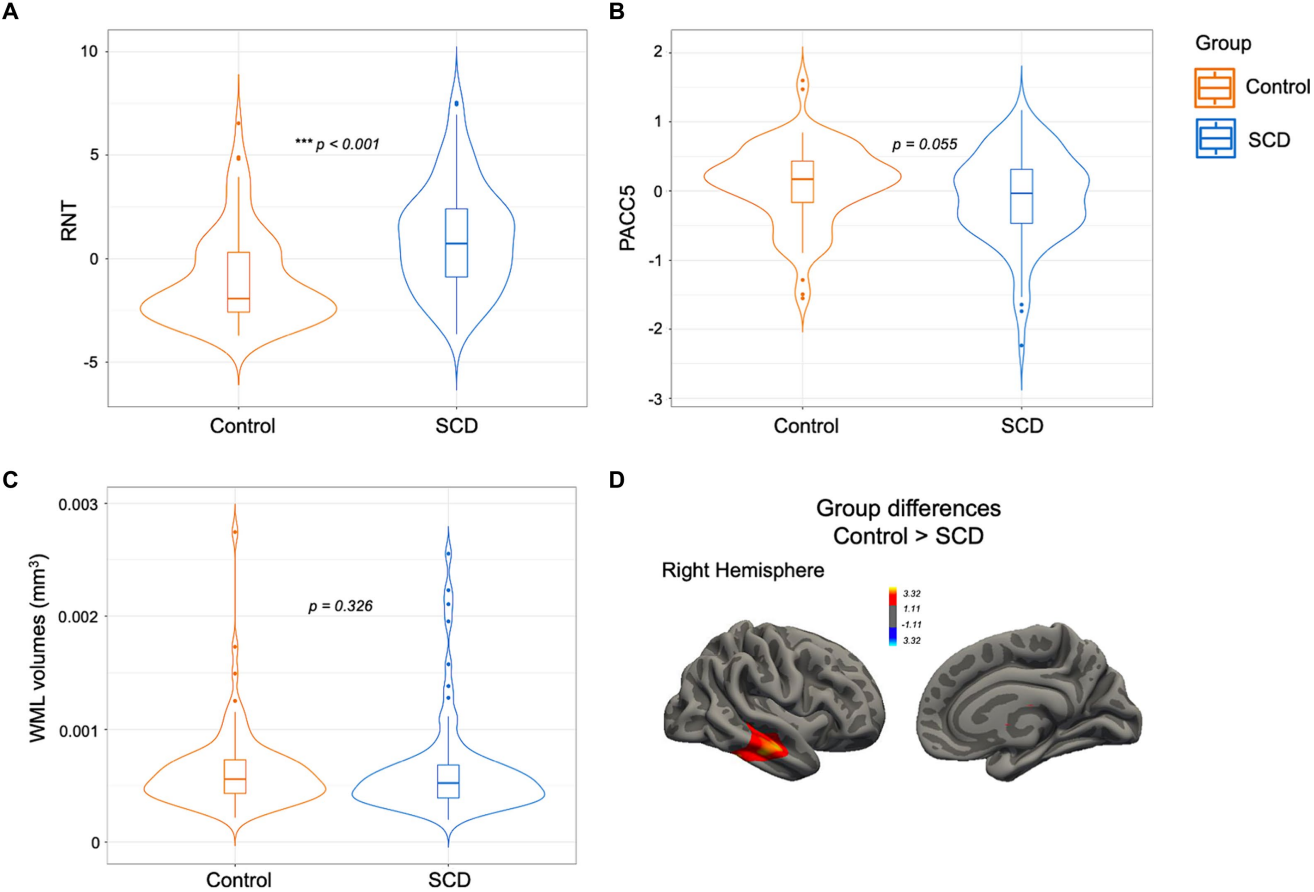
SCD vs. controls comparison for **(A)** RNT, **(B)** PACC5, and **(C)** WML volumes. The box plot overlaps with a violin plot displaying the distribution of the variables in both groups, where the horizontal line represents the group median **(A–C)**. In section **(D)** the brain cluster is represented over a standard surface and remained significant after multiple comparison corrections with a final cluster-wise *p* < 0.05. SCD, subjective cognitive decline; RNT, repetitive negative thinking; PACC5, preclinical Alzheimer cognitive composite; WML, white matter lesions. ^*^p < 0.05, ^**^p < 0.01, ^***^p < 0.001.

As per the establishment of our control group matched, there were no significant differences in age, sex, and educational level (*p* > 0.05). Regarding the first aim of the study about group differences, SCD participants showed higher rates of RNT (W = 2003, *p* < 0.001; see Figure 2A) and a trend toward lower PACC5 values (W = 4569.5, *p* = 0.055; see Figure 2B). There was no group difference in the volume of WML (W = 435, *p* = 0.326; see Figure 2C). In the MRI vertex-wise analyses after Monte Carlo correction for multiple comparisons, a significant cluster emerged in the right middle and inferior temporal regions (CWP = 0.009; see Figure 2D), where SCD individuals exhibited a thinner cortex compared to controls.

Results concerning associations between demographic variables, PACC5, RNT, and brain integrity are explained in Supplementary material.

### 3.2 Associations between PACC5 and brain integrity measures

**Figure 3:**
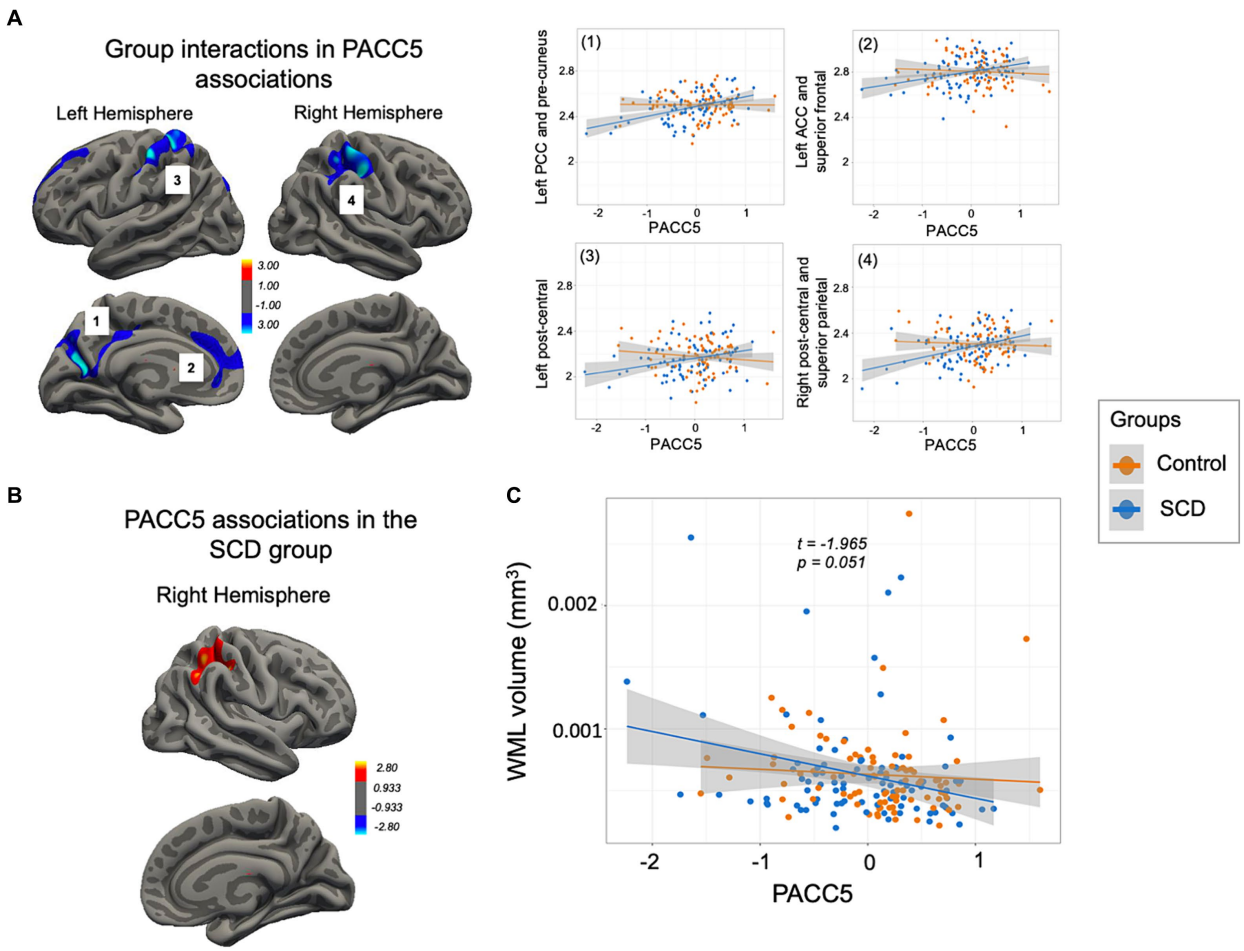
PACC5 anatomical correlates. **(A)** Significant clusters derived from the interaction analysis (multiple comparison corrections, final cluster-wise *p* < 0.05) comprising: (1) the left PCC, cuneus and pre-cuneus and superior parietal cortex (CWP = 0.005), (2) the left rostral ACC and superior frontal cortex (CWP = 0.010), (3) the left post-central cortex (CWP = 0.017), and (4) the right post-central and inferior and superior parietal cortex (CWP = 0.000). Individual mean CTh values were extracted for plotting group interaction (in the right). **(B)** Cluster encompassing the right post-central and inferior and superior parietal cortex shown in yellow (CWP = 0.002), where only the SCD group presented a positive association with PACC5. **(C)** Scatterplot showing a group interaction trend in the association between the PACC5 and WML volumes. SCD, subjective cognitive decline; CTh, cortical thickness; PACC5, preclinical Alzheimer cognitive composite; PCC, posterior cingulate cortex; ACC, anterior cingulate cortex; WML, white matter lesions.

In the whole-brain vertex-wise analyses, no relationship was found between CTh and PACC5 for the entire sample, but group interactions were observed in four brain clusters involving the frontal and parietal cortex (see Figure 3A for regions and CWP details). Subsequent analyses showed that greater right parietal CTh in the SCD group was positively associated with better PACC5 performance (see Figure 3B for regions and CWP details). In contrast, the control group did not display any significant association between CTh and PACC5.

WML volumes were not related to PACC5 in the entire sample (*β* = −0.000, SE = 0.000, *t* = −0.161, *p* = 0.872), but a trend toward a group interaction emerged (*β* = −0.000, SE = 0.000, *t* = −1.965, *p* = 0.051; see Figure 3C), suggesting a negative but non-significant relationship between PACC5 and WML volumes only in the SCD group (*p* > 0.05).

### 3.3 Associations between RNT, PACC5, and brain integrity measures

#### 3.3.1 Associations between RNT and PACC5

Within the entire sample, we observed a trend toward a negative association between RNT and PACC5 (*β* = −0.029, SE = 0.016, *t* = −1.789, *p* = 0.075). No group interaction was found (*p* > 0.05).

#### 3.3.2 Associations between RNT and brain integrity measures

**Figure 4:**
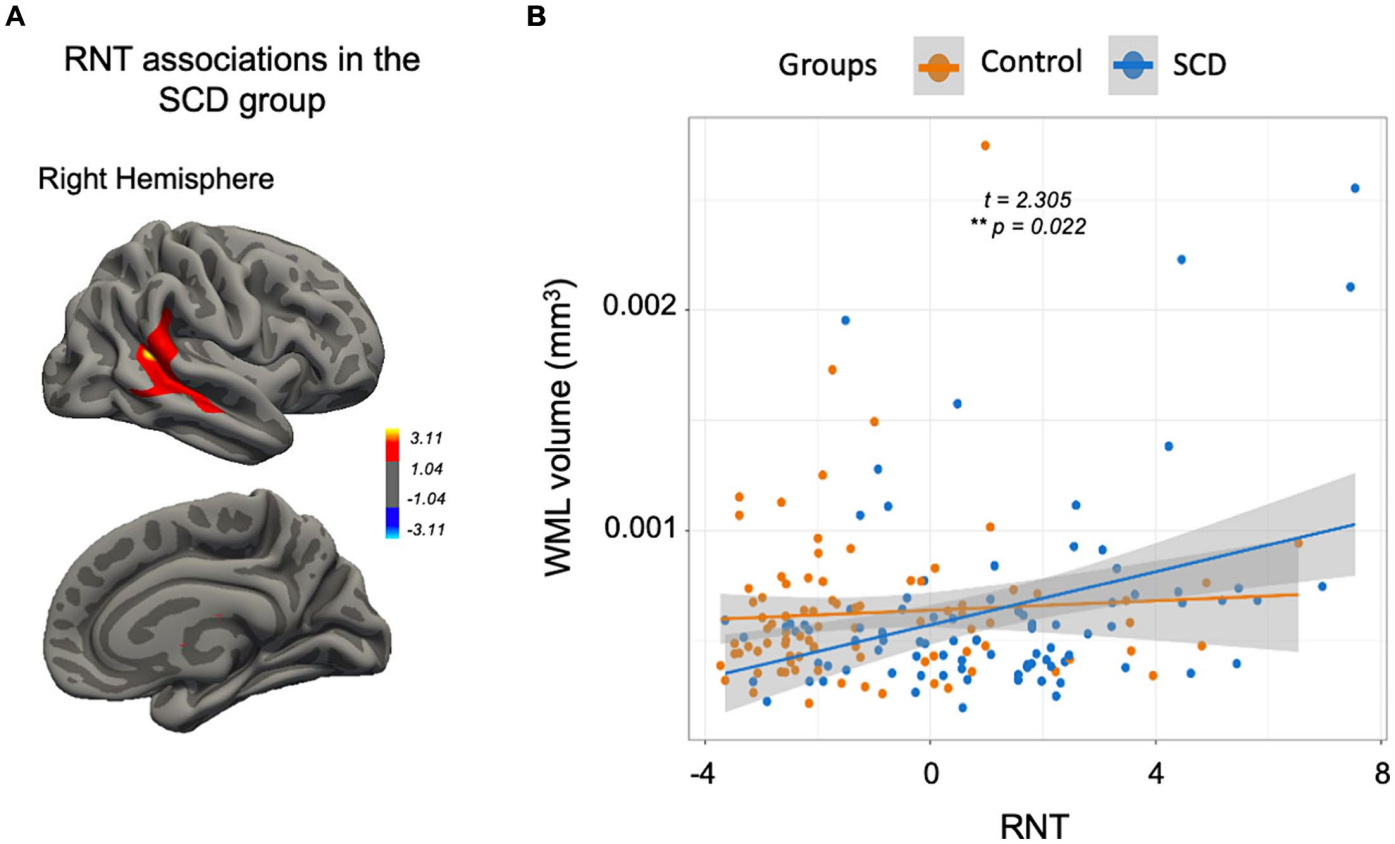
RNT correlates. **(A)** Significant cluster (multiple comparison corrections, final cluster-wise *p* < 0.05) where a positive association between RNT and the CTh was identified only in the SCD group. **(B)** The scatterplot displays a significant group interaction in the RNT-WML association where the SCD group evidenced a positive association. SCD, subjective cognitive decline; WML, white matter lesions; RNT, repetitive negative thinking; CTh, cortical thickness. ^*^p < 0.05, ^**^p < 0.01, ^***^p < 0.001.

For the entire sample, no significant associations were identified between RNT and CTh (*p* > 0.05). However, within the SCD group, greater RNT rates were positively correlated with higher CTh in the right supramarginal gyrus and the superior temporal sulcus (CWP = 0.010; see Figure 4A).

While no significant effect was observed between RNT and WML volumes for the entire sample (*β* = 0.000, SE = 0.000, *t* = 1.913, *p* = 0.058), we found a significant group interaction (*β* = 0.000, SE = 0.000, *t* = 2.305, *p* = 0.022) where the SCD group exhibited a positive relationship between RNT and WML volumes (*β* = 0.000, SE = 0.000, *t* = 2.806, *p* = 0.006), contrasting with the control group (see Figure 4B).

#### 3.3.3 Associations between PACC5, RNT, and brain integrity measures based on RNT levels by the SCD group

We conducted independent multivariate GLMs as exploratory analyses to investigate the relationships of CTh and WML within each subgroup of RNT level in the SCD group (i.e., highRNT-SCD and lowRNT-SCD). The descriptive statistics for the subgroups (Supplementary Table S2) and subgroup comparisons were defined in the Supplementary material.

**Figure 5:**
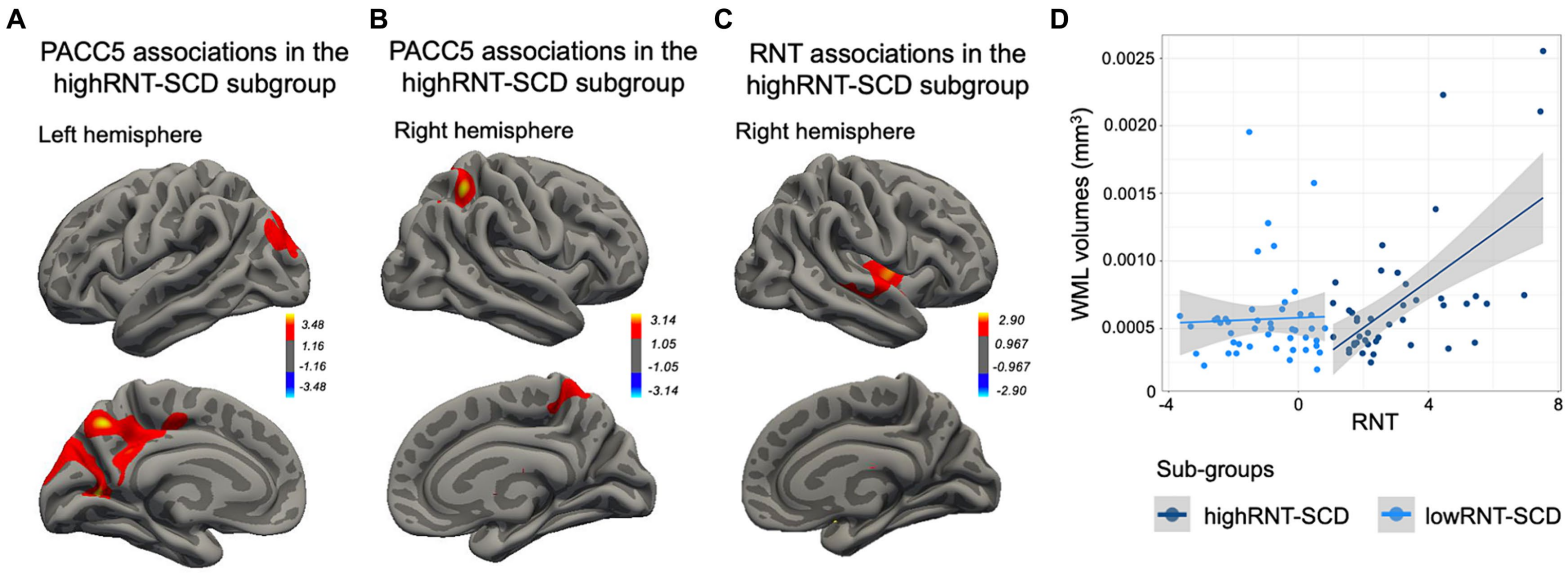
HighRNT-SCD subgroup correlates. **(A)** Positive correlations between PACC5 and CTh in two brain clusters in the left hemisphere, including (1) the PCC, lingual gyrus, and lateral occipital regions, and (2) the superior parietal, precuneus, cuneus, and isthmus cingulate regions. **(B)** A positive correlation between PACC5 and one brain cluster, comprising the right superior parietal and the precuneus regions. **(C)** A positive correlation between RNT values and a brain cluster comprising the right insula and superior temporal regions. **(D)** Scatterplot showing a positive association between WML volumes and RNT rates only in the highRNT-SCD subgroup. SCD, subjective cognitive decline; PACC5, preclinical Alzheimer cognitive composite; CTh, cortical thickness; RNT, repetitive negative thinking; WML, white matter lesions.

On the one hand, although no subgroup interactions were found, only in the highRNT-SCD subgroup better PACC5 performance was associated with higher CTh within two significant clusters in the left hemisphere, encompassing the posterior cingulate cortex (PCC), lateral occipital, superior parietal, precuneus and cuneus, lingual, and isthmus cingulate (CWP = 0.000 and 0.007; see Figure 5A), and one cluster in the right hemisphere comprising the superior parietal and precuneus (CWP = 0.035; see Figure 5B). Moreover, the highRNT-SCD subgroup showed a positive correlation between CTh and RNT in the right insula and superior temporal regions (CWP = 0.041; see Figure 5C).

On the other hand, neither highRNT-SCD nor lowRNT-SCD subgroups evidenced a significant association between WML and PACC5. Furthermore, although no subgroup interaction was found, only the highRNT-SCD subgroup demonstrated a significant positive association between WML and RNT values (*β* = 0.000, SE = 0.000, *t* = 2.425, *p* = 0.020; see Figure 5D).

## 4 Discussion

Our study revealed that community-based SCD individuals exhibited simultaneously higher RNT levels and thinner temporal CTh compared to the control group. However, we did not observe significant differences in global cognitive function or WML burden between the two groups. These findings suggest the complex nature of SCD, highlighting potential implications for early age-related changes in grey matter rather than white matter. Our investigation also sought to explore the relationships among RNT, global cognition, and grey and white matter integrity in the SCD condition compared to a control state. We were able to report, for the first time, the relationships between RNT and brain integrity (both grey and white matter) in a healthy sample of middle-aged to older SCD individuals.

Our results align with previous studies with SCD, showing associations with RNT rates (Schlosser et al., 2020) and reduced temporal CTh in SCD groups (Jessen et al., 2020; Rivas-Fernández et al., 2023). Verfaillie et al. (2016) and Lim et al. (2019) demonstrated that SCD individuals who progressed to MCI, clinical AD, or non-AD dementia within 3–5 years had thinner baseline temporal and parietal cortices, part of the AD signature (Dickerson and Wolk, 2012). Moreover, community-based SCD individuals often exhibit greater cortical atrophy in temporal regions compared to clinical-based samples, which show atrophy in frontal and insular regions associated with depression and anxiety symptoms (see reviews Pini and Wennberg, 2021; Arrondo et al., 2022). The main difference between community-based and clinical-based SCD samples is that the former do not seek clinical help, while the latter visit a physician due to cognitive complaints. Despite similar AD conversion rates among the community and clinical-based SCD samples (Mitchell et al., 2014), Pini and Wennberg (2021) suggested that discrepancies in the cortical atrophy pattern results rely on the lower awareness in SCD individuals who do not seek help, as anosognosia is a classic feature of dementia (Hanseeuw et al., 2020). In contrast, the clinical samples are more heterogeneous due to the potential psychiatric background of their cognitive complaints (Espenes et al., 2020). However, prior research observed higher WML volumes in SCD groups and positive correlations between cognitive complaints and WML burden (Van Rooden et al., 2018; Morrison et al., 2023), but our study in middle-aged and older adults found the same WML burden between SCD and control groups. This discrepancy in results may be attributed to the reason that WML, representing a broader finding within the aging population, may be less specific to SCD, while CTh offers high sensitivity for detecting structural early changes in SCD.

Moreover, group interactions in four brain clusters indicated that the PACC5, previously defined as a preclinical dementia marker (Papp et al., 2017), had enough sensitivity to detect early CTh variations in the SCD condition compared to controls. The detected cortical regions positively related to PACC5 in SCD and interacting with controls, namely the PCC, precuneus, cuneus, parietal and frontal regions, and the anterior cingulate cortex (ACC), have been previously associated with early age-related cognitive changes (McDonald et al., 2009) and greater amyloid-B burden in SCD patients with higher rates of worry (Schwarz et al., 2021). On the other hand, while other studies on SCD have demonstrated that individuals with a higher burden of WML tend to have poorer cognitive status (Rothstein et al., 2023), we could not differentiate the association between PACC5 and WML among our SCD and control groups due to a lack of group interaction. Although our SCD participants tended to have lower PACC5 scores as WML increased, the association was not significant. This suggests that WML burden, at least in the range observed in the present study, may not be sufficient to negatively impact cognition.

In the literature, RNT has evidenced both positive and negative associations with CTh and regional grey matter volume mostly in prefrontal and cingulate regions (Demnitz-King et al., 2021). Demnitz-King et al. (2021) concluded that pathological levels of RNT in clinical populations may be associated with maladaptive outcomes in terms of reduced grey matter volumes, while moderated levels of RNT in healthy groups might have positive associations reflecting increased use of certain brain regions due to persistent high cognitive demands. In our work, we found only in SCD individuals a positive relationship between RNT and parietotemporal areas including the right supramarginal gyrus, and the superior and middle temporal gyri only in SCD individuals. Although SCD is considered a preclinical stage of AD, it is characterized by individuals who do not exhibit cognitive impairment or psychiatric comorbidity. Consequently, a positive association in our sample could be aligned with this perspective. Likewise, Demnitz-King et al. (2021) also suggested that inconsistent RNT associations in the literature as regards specific brain areas emerged likely due to the diffuse nature of ruminative thoughts supported by distributed brain regions rather than a single structure (also depending on the thoughts’ content). Our findings, pointing to an association within the supramarginal gyrus in our SCD individuals, align with the role of this area in sustaining emotion regulation ability in the general population (Wada et al., 2021). In addition, the supramarginal gyrus is a brain region vulnerable to psychiatric disorders such as major depression (Suh et al., 2019), a disorder where RNT is a transdiagnostic factor (Spinhoven et al., 2018). However, in our non-clinical population, the positive association between moderate levels of RNT and the supramarginal gyrus may reflect the functional use of this brain region in emotion regulation and cognitive processes.

Besides, we found that only in the SCD group there was a positive impact of RNT on WML, indicating that those individuals with higher RNT rates presented more WML burden. Microstructural abnormalities in white matter integrity in long-range fasciculi have been previously linked to RNT in both clinical and non-clinical populations (Demnitz-King et al., 2021). Moreover, previous studies in SCD have shown that individuals with a higher burden of WML (Rothstein et al., 2023) are more likely to be amyloid-positive (Hong et al., 2019), and may progress to MCI or dementia (Benedictus et al., 2015). These findings, along with our results, potentially position RNT as a risk factor for subsequent age-related pathological processes in SCD.

Further supporting this notion, our exploratory analyses show that the brain structures that emerged as positively associated with cognition and RNT included the PCC and the insula, respectively, in the SCD subgroup with higher RNT. A recent publication by Solé-Padullés et al. (2022) reported that while the RNT was related to cognitive complaints in a healthy sample, it predicted the segregation of the anterior salience resting-state network, with key hubs including the ACC and insula, which seem to be vulnerable in SCD (Xue et al., 2021). Thus, in our study, it is suggested that RNT impacts the function and structure of the insula, and consequently the salience network. Those structures play crucial roles in responding to salient stimuli and generating emotional responses, which pose challenges to cognitive demands (Menon and Uddin, 2010). These regions are also considered structurally and functionally preserved in successful aging (de Godoy et al., 2021). As regards white matter integrity, even though no group interaction was found, also only the SCD individuals within the high RNT subgroup showed a positive association between RNT and WML volumes.

The present study has some limitations. First, we included individuals meeting SCD criteria derived from a community-based sample, which may limit the direct applicability of our findings to clinical populations. Second, there is a diverse range of SCD criteria in the literature (i.e., SCD and SCD-plus), which warrants caution when replicating and comparing results across studies. Finally, this was a cross-sectional study and future research should prioritize longitudinal investigations to elucidate the temporal sequence of cognitive decline, and brain status within the context of SCD and RNT. Additionally, exploring interventions targeting RNT may offer promising avenues for mitigating cognitive decline and preserving brain health in at-risk populations such as SCD.

## 5 Conclusion

Our study found that individuals with SCD, even in the middle to older ages, had higher RNT levels and reduced CTh in the right temporal cortex compared to controls. Only in the SCD group, global cognitive status was associated with CTh rather than WMLs, and RNT was positively related to both brain integrity measures. These findings highlight the importance of considering modifiable psychological factors, such as RNT, alongside traditional neurobiological markers when studying neurocognitive status in SCD populations. While our findings deepen the understanding of the complex relationship between psychological factors, cognitive function, and brain structure in SCD, insufficient evidence exists to conclusively determine how RNT influences the connection between cognition and brain integrity.

## Data availability statement

The datasets presented in this article are not readily available because data, analytic methods, and study materials will not be available to other researchers for the moment. Requests to access the datasets should be directed to dbartres@ub.edu and lidiavaque@ub.edu.

## Ethics statement

The studies involving humans were approved by the Institutional Review Board (IRB 00003099 at the University of Barcelona) and Comité d’Ètica i Investigació Clínica de la Unió Catalana d’Hospitals. The studies were conducted in accordance with the local legislation and institutional requirements. The participants provided their written informed consent to participate in this study.

## Author contributions

LM-P: Conceptualization, Data curation, Formal analysis, Investigation, Methodology, Visualization, Writing – original draft, Writing – review & editing. CS-P: Conceptualization, Investigation, Supervision, Writing – review & editing. MC-T: Data curation, Formal analysis, Methodology, Project administration, Software, Writing – review & editing. KA-P: Project administration, Writing – review & editing. RP-A: Project administration, Writing – review & editing. GC: Data curation, Funding acquisition, Project administration, Resources, Validation, Writing – review & editing. JS: Data curation, Funding acquisition, Project administration, Resources, Validation, Writing – review & editing. VA-S: Data curation, Project administration, Writing – review & editing. NB: Data curation, Resources, Writing – review & editing. JT-M: Writing – review & editing, Funding acquisition, Project administration, Resources, Validation. AP-L: Funding acquisition, Project administration, Resources, Validation, Writing – review & editing. DB-F: Conceptualization, Data curation, Formal analysis, Funding acquisition, Investigation, Methodology, Project administration, Resources, Supervision, Validation, Writing – review & editing. LV-A: Formal analysis, Investigation, Methodology, Supervision, Validation, Writing – review & editing, Conceptualization, Data curation.

## Footnotes

^1^http://surfer.nmr.mgh.harvard.edu

^2^http://www.posit.co/

^3^https://gking.harvard.edu/matchit

^4^http://www.rstudio.com/
