# Supplementary material for: Impact of repetitive negative thinking on subjective cognitive decline: insights into cognition and brain structure
Source: Front Aging Neurosci. 2024 Aug 13;16:1441359. doi: 10.3389/fnagi.2024.1441359 (PMC11347316; doi:10.3389/fnagi.2024.1441359)
Supplement: Supplementary file 1 [file Data_Sheet_1.docx]

**Supplementary Material**

**INDEX**

MRI acquisition and preprocessing ………………………………………….. Pag. 2

DESCRIPTION OF OUR GROUPS OF STUDY …………………………… Pag. 3

Table S1. Descriptive statistics for the entire sample and SCD and control groups ………………………………………………………………………………..….. Pag. 3

ADDITIONAL RESULTS ……………………………………………….……… Pag. 3

Additional results in demographics and group differences …………...…… Pag. 3

Figure S1. CTh relationships with age for the entire sample ……………… Pag. 4

Additional results in brain integrity associations as regards RNT levels in the

SCD group ………………………………………………………………….…… Pag. 5

Table S2. Descriptive statistics for the subgroups highRNT-SCD and

lowRNT-SCD …………………………………………………………………… Pag. 5

REFERENCES ………………………………………………………………… Pag. 6

**MRI acquisition and preprocessing**

For all participants, a high-resolution T1-weighted structural image was obtained with a magnetization-prepared rapid acquisition gradient-echo (MPRAGE) three-dimensional protocol [repetition time (TR) = 2,400 ms, echo time (TE) = 2.22 ms, inversion time = 1,000 ms, field of view (FOV) = 256 mm, 0.8-mm isotropic voxel]. Additionally, a high-resolution 3D SPC T2-weighted structural brain MRI was undertaken (TR = 3,200 ms, TE = 563 ms, flip angle = 120º, 0.8 mm isotropic voxel, FOV = 256 mm). MRI images were examined by a senior neuroradiologist [N.B] to discard any clinically significant pathology. Additionally, all acquisitions were visually inspected before analysis by two co-authors [L.M.-P and M.C.-T] to ensure that they did not contain MRI artifacts or excessive motion.

Automated preprocessing of structural T1-weighted images was carried out using FreeSurfer (version 6.0, http://surfer.nmr.mgh.harvard.edu). CTh maps were generated as the distance between the white and grey matter surfaces at each vertex of the reconstructed cortical mantle (Fischl et al., 2002). Initially, the images were processed individually using contrasted T2-weighted images to improve pial surfaces, and the results were visually reviewed to confirm the accuracy of registration, skull stripping, segmentation, and cortical surface reconstruction. Before the statistical analysis, CTh maps were smoothed using a

2D Gaussian kernel of 15 mm full width at half maximum (FWHM).

After T1-weighted automated preprocessing and segmentation using FreeSurfer,

the total WML burden of the whole brain was quantified as the total volume (in mm^3^) of all voxels identified as white matter hypointensities in the standard space.

Subsequently, we adjusted for head size by estimating the ratio of WML volume to the estimated total intracranial volume. The hypointensities demonstrated strong correlations with hyperintensities measured by the T2/FLAIR sequence and with other markers of cerebral vessel disease, such as the Fazekas score (Cedres et al., 2020).

To study group differences in CTh and the associations with CTh, we carried out whole-brain vertex-wise General Linear Models (GLMs) in FreeSurfer. Moreover, all CTh vertex-wise analyses were corrected for family-wise error (FWE) using a Monte Carlo Null-Z simulation with 10,000 repetitions and a cluster-wise p-value (CWP) p < 0.05.

**DESCRIPTION OF OUR GROUPS OF STUDY**

**Table S1.** Descriptive statistics for the entire sample and SCD and control groups

|  | Whole sample (N=178)  *Mean ± SD* | SCD group  (N=89)  *Mean ± SD* | Control group  (N=89)  *Mean ± SD* |
| --- | --- | --- | --- |
| Age | 56.13 ± 7.72 | 56.18 ± 7.77 | 56.09 ± 7.75 |
| Sex | 122 females (68.54%) | 61 females (68.54%) | 61 females (68.54%) |
| Educational level | 8 primary (4.49%) 47 secondary (26.40%) 123 tertiary (69.10%) | 4 primary (4.49%) 24 secondary (26.97%) 61 tertiary (68.54%) | 4 primary (4.49%) 23 secondary (25.84%) 62 tertiary (69.66%) |
| PSWQ | 13.70 ± 12.00 | 17.53 ± 12.52 | 9.86 ± 10.16 |
| PTQ | 17.92 ± 12.21 | 22.44 ± 11.96 | 13.38 ± 10.72 |
| RRS | 15.50 ± 12.06 | 19.81 ± 12.40 | 11.19 ± 10.06 |
| RNT | 0.00 ± 2.61 | 1.05 ± 2.55 | -1.05 ± 2.22 |
| PACC5 | 0.00 ± 0.65 | -0.10 ± 0.67 | 0.10 ± 0.61 |

Abbreviations. SCD, subjective cognitive decline; SD, standard deviation; PSWQ, perseverative state worry questionnaire; PTQ, perseverative thinking questionnaire; RRS, ruminative response scale; RNT, repetitive negative thinking; PACC5, preclinical Alzheimer cognitive composite 5.

**ADDITIONAL RESULTS**

1. **Additional results in demographics and group differences**

Additionally, we conducted general linear models (GLMs) to explore whether demographics influenced preclinical Alzheimer’s cognitive composite (PACC5) and repetitive negative thinking (RNT). Age exhibited a negative association with PACC5 (β = -0.044, SE = 0.005, t = -8.202, p < 0.001), while individuals with a tertiary level of education demonstrated higher PACC5 scores (β = 0.456, SE = 0.196, t = 2.340, p = 0.024) compared to those with a secondary and primary level of education. No significant relationship was found between PACC5 and sex. On the other hand, RNT was positively related to age (β = 0.091, SE = 0.024, t = 3.687, p < 0.001), but no significant associations were found with sex or educational level (p > 0.05).

To ensure that our measures of brain integrity accurately reflected the process of brain aging, we investigated the associations between both cortical thickness (CTh) and white matter lesions (WML) volumes and age. Whole-brain vertex-wise analyses revealed negative correlations between age and bilateral CTh in regions such as the superior and inferior parietal, the lateral occipital, the insula, the banks of the superior temporal sulcus, the supramarginal, the precentral, the rostral middle and superior frontal, the superior, middle, and medial temporal (including entorhinal, fusiform, parahippocampal and lingual) and cuneus and precuneus (clusterwise p-values [CWP], left = 0.000 and right = 0.000; see Figure S1). Cingulate regions (the rostral, anterior, posterior, and isthmus cingulate cortex) exhibited negative correlations with age only in the left hemisphere. Furthermore, WML volumes showed a negative correlation with age (Spearman, rho = 0.417, p < 0.001).


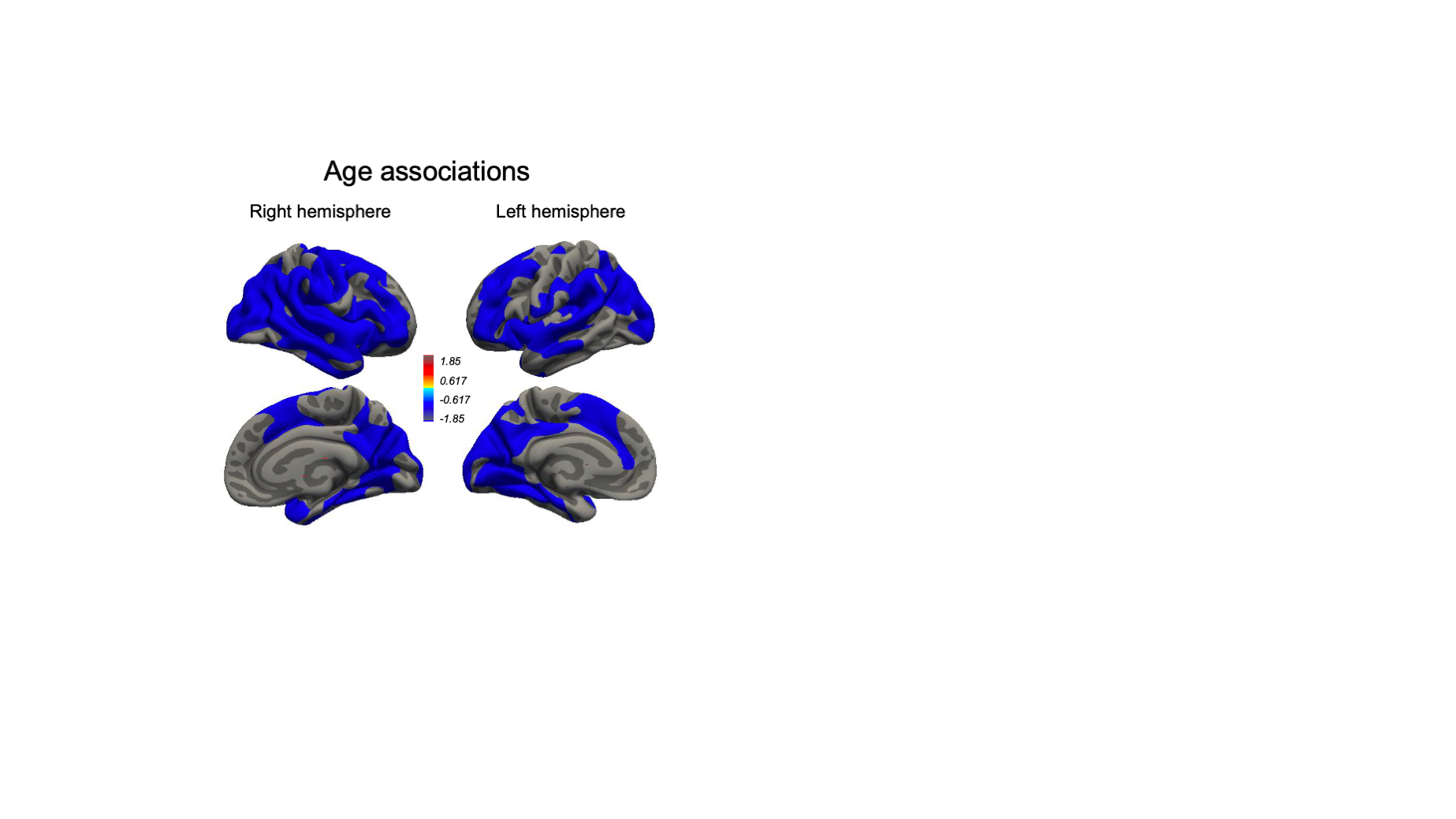


**Figure S1.** CTh relationships with age for the entire sample (N = 178). Significant brain clusters from vertex-wise whole-brain analyses, illustrating CTh relationships with age for the entire sample. Negative bilateral correlations were observed across a wide area. All clusters are represented over a standard surface and remained significant after multiple comparison corrections with a final cluster-wise p < 0.05. Abbreviations: CTh, cortical thickness.

**Additional results in brain integrity associations as regards RNT levels in the SCD group**

Based on RNT scores at the 50th percentile (median = 0.738), we categorized SCD participants into two subgroups: lowRNT-SCD (RNT values ≤ 0.738, N = 45) and highRNT-SCD (RNT values > 0.738, N = 44). Then, as complementary analyses, we conducted independent multivariable GLMs to explore the associations of CTh and WML volumes within the subjective cognitive decline (SCD) group based on levels of RNT (lowRNT-SCD and highRNT-SCD subgroups).

Firstly, we compared the two subgroups in terms of demographics and the main variables of interest in the study, as presented in Table S2.

Both subgroups based on RNT levels did not exhibit differences in age (W = 1106.5, p = 0.247), sex (X^2^ = 1.551, p-value = 0.213), or educational level (X^2^ = 0.362, p = 0.834). Our groups showed akin values in PACC5 (W = 891.5, p = 0.529) and WML volumes (W = 1158, p = 0.113), with no differences in CTh.

**Table S2.** Descriptive statistics for the subgroups highRNT-SCD and lowRNT-SCD

|  | LowRNT-SCD subgroup (N=45)  *Mean ± SD* | HighRNT-SCD subgroup (N=44)  *Mean ± SD* |
| --- | --- | --- |
| Age | 55.11 ± 7.18 | 57.33 ± 8.29 |
| Sex | 28 females (62.22%) | 33 females (75%) |
| Educational level | 2 primary (4.44%) 13 secondary (28.89%) 30 tertiary (66.67%) | 2 primary (4.54%) 11 secondary (25%) 31 tertiary (70.46%) |
| PSWQ | 9.02 ± 5.55 | 26.21 ± 11.80 |
| PTQ | 14.67 ± 9.55 | 30.37 ± 8.54 |
| RRS | 11.33 ± 6.50 | 28.21 ± 10.91 |
| RNT | -1.00 ± 1.21 | 3.12 ± 1.73 |
| PACC5 | -0.04 ± 0.58 | -0.14 ± 0.72 |

Abbreviations. SCD, subjective cognitive decline; SD, standard deviation; PSWQ, perseverative state worry questionnaire; PTQ, perseverative thinking questionnaire; RRS, ruminative response scale; RNT, repetitive negative thinking; PACC5, preclinical Alzheimer cognitive composite 5.

**REFERENCES**

Fischl, B. *et al.* (2002). Whole Brain Segmentation: Neurotechnique Automated Labeling of Neuroanatomical Structures in the Human Brain. *Neuron*, *33*, 341–355. <https://doi.org/10.1016/s0896-6273(02)00569-x>.

Cedres, N. *et al.* (2020). Predicting Fazekas scores from automatic segmentations of white matter signal abnormalities. *Aging*, 12(1), 894–901. https://doi.org/10.18632/aging.102662
